# Urban Trees and Hydrological Ecosystem Service: A Novel Approach to Analyzing the Relationship Between Landscape Structure and Runoff Reduction

**DOI:** 10.1007/s00267-023-01868-z

**Published:** 2023-08-26

**Authors:** Vahid Amini Parsa, Mustafa Nur Istanbuly, Jakub Kronenberg, Alessio Russo, Bahman Jabbarian Amiri

**Affiliations:** 1 Social-Ecological Systems Analysis Lab, Faculty of Economics and Sociology, University of Lodz, Lodz, Poland; 2 Department of Natural Resources and Environment, University of Aleppo, Aleppo, Syria; 3 School of Arts, University of Gloucestershire, Cheltenham, UK; 4 Department of Regional Economics and the Environment, Faculty of Economics and Sociology, University of Lodz, Lodz, Poland

**Keywords:** Regulating ecosystem services, Ecosystem service modelling, Green infrastructure, Landscape ecology, Landscape metrics, Urban water management

## Abstract

Urban stormwater runoff has posed significant challenges in the face of urbanization and climate change, emphasizing the importance of trees in providing runoff reduction ecosystem services (RRES). However, the sustainability of RRES can be disturbed by urban landscape modification. Understanding the impact of landscape structure on RRES is crucial to manage urban landscapes effectively to sustain supply of RRES. So, this study developed a new approach that analyzes the relationship between the landscape structural pattern and the RRES in Tabriz, Iran. The provision of RRES was estimated using the i-Tree Eco model. Landscape structure-related metrics of land use and cover (LULC) were derived using FRAGSTATS to quantify the landscape structure. Stepwise regression analysis was used to assess the relationship between landscape structure metrics and the provision of RRES. The results indicated that throughout the city, the trees prevented 196854.15 m^3^ of runoff annually. Regression models (*p* ≤ 0.05) suggested that the provision of RRES could be predicted using the measures of the related circumscribing circle metric (0.889 ≤ *r*^*2*^ ≤ 0.954) and the shape index (*r*^*2*^ = 0.983) of LULC patches. The findings also revealed that the regularity or regularity of the given LULC patches’ shape could impact the patches’ functions, which, in turn, affects the provision of RRES. The landscape metrics can serve as proxies to predict the capacity of trees for potential RRES using the obtained regression models. This helps to allocate suitable LULC through optimizing landscape metrics and management guidance to sustain RRES.

## Introduction

Global unrestrained urbanization alters urban natural ecosystems and landscape structure and increases the share of impermeable surfaces in cities (Mullaney et al. 2015; Senes et al. 2021). This greatly leads to modification and disruption of the urban hydrological cycle, resulting in increased magnitude of surface water runoff and local flooding (Xu et al. 2013; Qian and Eslamian 2022). This issue is further accelerated by the extreme weather events due to global climate change in cities (Kumar et al. 2022; Muyambo et al. 2023). Consequently, not only does excessive stormwater runoff increase, but also the capability of cities to deal with these challenges diminishes (McGrane 2016; Janke et al. 2017; Zhou et al. 2021). Urban stormwater can seriously affect ecosystems, built environment, people, and property (Beck et al. 2016; Subramanian 2017).

Traditional stormwater management approaches (gray infrastructure) are often inadequate and unsustainable to mitigate the current and future impacts and are also expensive to construct and maintain (US EPA 2017; Lu and Wang 2021). This has led to a demand for alternative and complementary cost-effective and sustainable approaches, primarily involving urban green infrastructure (UGI) (Wang et al. 2008; Carlyle-Moses et al. 2020; Hamel and Tan 2022). This highlights the importance of providing hydrologic ecosystem services (HES) by UGI in general and provision of runoff reduction by urban trees (RRES) in particular to mitigate stormwater issues. Urban trees facilitate HES and interact with the urban hydrologic cycle (Szota et al. 2018; Van Stan et al. 2020). The HES can decrease flow rate, peak runoff, and flooding hazards (Xiao and McPherson 2002; Kermavnar and Vilhar 2017). Previous studies have shown the positive effects of UGI, specifically urban trees, on surface runoff (Asadian and Weiler 2009; Inkiläinen et al. 2013; Li et al. 2020; Liu et al. 2020).

The sustainability of HES is disturbed by urban landscape modification (Qiu and Turner 2015; Duarte et al. 2018; Metzger et al. 2021). Hydrological characteristics of a given area, including but not limited to water flow, are more influenced by landscape structure (shape or form) (Uuemaa et al. 2007). Changes in the urban spatial landscape structure alter ecological (ecosystem) functions, processes, and flow patterns (Mitchell et al. 2013; Muleta and Biru 2019). This, in turn, substantially alters the capability of urban ecosystems to provide various ecosystem services (ES), either positively or negatively (Chen et al. 2021; Yohannes et al. 2021). It is crucial to the regulating ES, particularly HES, as their supply, demand, and flow are explicitly linked to the movement and flow of the matter across urban landscapes (Eigenbrod 2016; Xia et al. 2021).

Increasing evidence, including theories (Mitchell et al. 2015a), meta-analysis (Mitchell et al. 2013; Duarte et al. 2018), conceptual frameworks (Inkoom et al. 2018), and case studies (Syrbe and Walz 2012; Kim and Park 2016; Duflot et al. 2017) has highlighted the impact of landscape structures on different ES, mainly in natural contexts. However, our understanding in this area is still in its early stages, and for many ES, how different aspects of landscape structures (most) affect their provision has not yet been well understood empirically (Lamy et al. 2016; Herrero-Jáuregui et al. 2019; Tran et al. 2022). These relations in cities are even more unclear due to the high complexity and heterogeneity (LaPoint et al. 2015; Grafius et al. 2016) and the lack of empirical studies (Dobbs et al. 2014; Grafius et al. 2018). Therefore, overcoming this critical knowledge gap in urban areas is essential.

Understanding what features of urban landscape structure affect the provision of ES, especially HES, substantially improves the landscape management knowledge and practices for sustainable ES provision (Breuste et al. 2013; Mitchell et al. 2015b). Based on the shape-function relationship, the patch’s geometrical and morphometric shape features (landscape structure) affect the landscape function regarding water flow (Amiri et al. 2019; With 2019). Landscape structural patterns are a dominant element of landscape structure (Karimi et al. 2021). It is considered a useful lever to affect the movement, flow, interaction, and provision of HES (Rieb and Bennett 2020). Although landscape structure is expected to significantly influence the provision of HES, it has not been widely studied in the urban context. Previous studies have appreciated the effects of urban landscape structure on some aspects of stormwater management through HES, including sediment erosion, flood control, peak runoff, freshwater supply, and surface and groundwater quality (Qiu and Turner 2015; Kim and Park 2016; Grafius et al. 2018; Inkoom et al. 2018; Metzger et al. 2021; Luo et al. 2022). Also, the impacts of LULC changes on runoff reduction ES have been acknowledged using landscape metrics (Zhang et al. 2015; Li et al. 2020). These studies primarily concentrated on mitigating runoff through ES provided by various LULC classes, specifically UGI, and relied on empirical models and runoff reduction coefficients from prior research to estimate the capacity of UGI to reduce runoff. However, an overlooked aspect in these studies is investigating the effects of landscape structural patterns on RRES. The existing literature highlights a gap in scientific understanding and empirical evidence concerning the relations between multiple measures of landscape structural patterns and provision of RRES, which is essential for developing ES-based landscape management tools to sustain RRES. To address this gap, this paper aims to analyze the role of urban landscape structure in the provision of RRES by analyzing the relations between landscape structural patterns and RRES in Tabriz, Iran. This city faces frequent heavy stormwater runoff and floods due to rapid urbanization, local climate and topography conditions, and global climate change, leading to severe flooding in densely inhabited areas (Mahmood Zadeh et al. 2015; Yazdani et al. 2018). Consequently, Tabriz was selected as the case study for scientific and practical purposes.

This paper seeks to empirically understand how RRES responds to the multiple measures of landscape structural pattern. The specific objectives were to (1) quantify the capacity of urban trees for runoff reduction, (2) quantify the measures of urban landscape structural pattern of LULC classes using landscape shape metrics, and (3) analyze the relations between the several measures of urban landscape structural pattern and the provision of RRES. The findings spur our understanding of how landscape structural pattern can influence the provision of RRES and help improve ES-based landscape management guidance to sustain RRES and more effectively manage stormwater runoff in cities.

## Materials and Methods

### Study Area

**Fig. 1:**
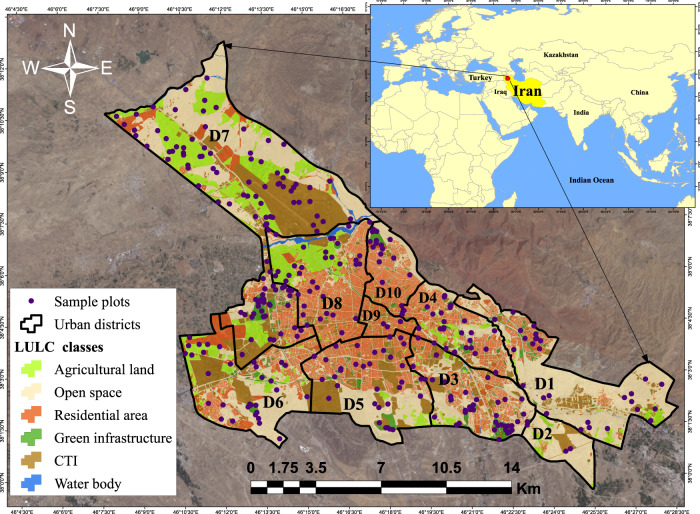
Location of the studied area, LULC classes, administrative districts, and sample plots. D1-D10 are urban districts

This study was conducted in Tabriz, the largest city in northwest Iran (Fig. 1). It has a population of about 1.56 million people and a 243 km² area (Statistical Center of Iran 2016). Tabriz has a mountainous topography (Asakereh and Akbarzadeh 2017), with a cold and semi-arid climate (Ghazi and Jeihouni 2022). The annual mean precipitation is 311.1 mm, with ~77.07 days experiencing rainfall of 1.0 mm or more (rainy days). The rainfall period is about 7.5 months, from 17 October to 1 June, with April having the highest average rainfall of 23 mm and August having the lowest average rainfall of 3 mm (IMO 2022). The rainfall pattern observed in Tabriz exhibits characteristics similar to that of the Mediterranean type (Jani1 et al. 2013). However, global climate change has affected the seasonal precipitation patterns, resulting in more intense rainfall events (Sanikhani et al. 2014; Sadeqi and Dinpashoh 2019). Tabriz faces a significant flood risk, with approximately 50% of its residents vulnerable to floods (Yazdani et al. 2018). The historical data showed that Tabriz had experienced about 42 cases of urban flooding from 1954 to 2009, resulting in significant human and economic losses (Soleimani-Alyar et al. 2016). Over the past century, rapid urban development and landscape changes have led to an increased share of impervious surfaces at the expense of decreasing green spaces (pervious surfaces) (Rahimi 2016).

### Data Sources

The administrative map and the initial LULC map for 2020 (scale 1:25000 and minimum mapping unit of 1 m) were obtained from the municipality of Tabriz. Hourly precipitation data of the synoptic station of Tabriz for a complete calendar year was received from the Iran Meteorological Organization (IMO 2022). Other meteorological data for executing the i-Tree Eco model were automatically retrieved from the archived NOAA database (Hirabayashi and Endreny 2016). Urban tree structural data were collected through the fieldwork.

### Methods

**Fig. 2:**
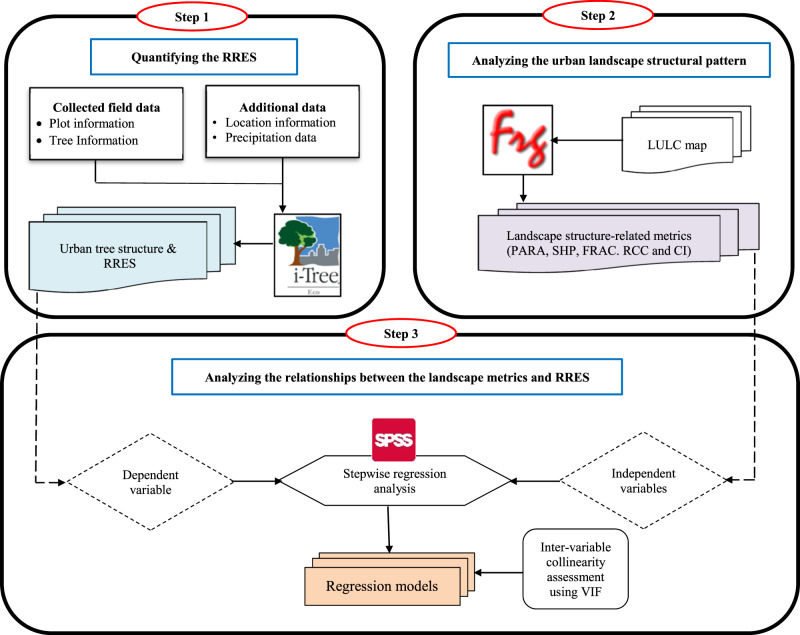
Summary of the methodological process

This study was carried out based on Fig. 2. The overall methodological approach of this study includes three main steps:

#### Assessing the Provision of RRES

To assess the provision of RRES, i-Tree tools were applied due to being one of the most appropriate, robust, fast, and process-based models to estimate RRES (Hirabayashi 2013; US EPA 2017; Nowak 2021). The i-Tree Eco model, exclusively developed for the U.S., was adapted for the study area by providing location information and hourly precipitation data to the i-Tree Database, following the protocol (i-Tree Eco International Projects 2016). The submitted data underwent a rigorous evaluation process by the U.S. Forest Service and was subsequently incorporated into the i-Tree Eco software. Subsequently, the recently appended location (Tabriz) was integrated into the subsequent versions of i-Tree Eco.

The required structural data for trees, including total height, live crown height, height to crown base, crown width, missing and health, species, tree cover, and diameter at breast height (DBH), were collected from 325 standard plots (with a radius of 11.34 m) through fieldwork during the leaf-on season following the manuals (i-Tree Eco User Manual 2016; i-Tree Field Guide 2016). Furthermore, the detailed data was collected for each plot, encompassing its precise geographical location and exact central coordinates, the proportion of the plot that was accessible and surveyed by the field crew, the percentage of the plot area covered by trees and shrubs, the quantity of space suitable for tree planting, identification of the reference objects from the plot center, the specific land use type within each plot, and the classification of ground cover types observed within each plot.

**Fig. 3:**
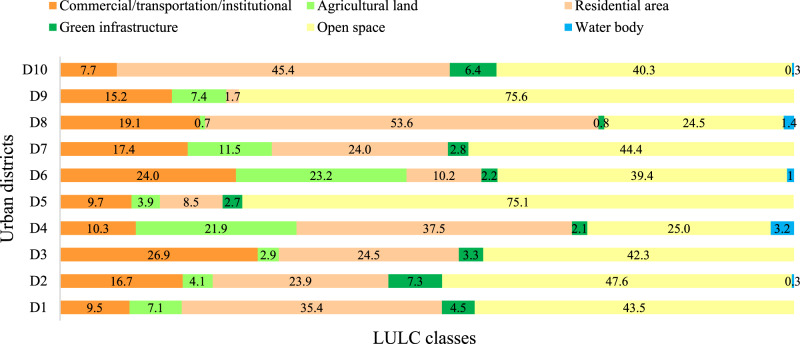
Area (%) of LULC types within each urban district (D1 (District 1) to D10 (District 10))

The sample size was chosen to balance data uncertainty, time constraints, limited resources, and costs for the field survey and achieve a standard error of approximately 10% for the entire city (Nowak et al. 2008). A unique methodological approach was employed to clarify the variations in RRES provision across the city. The plots were pre-stratified randomly among the LULC classes within the ten administrative districts to bring the multiple elements of RRES to each district and identify how the RRES provision varies across the districts. This approach was applied to obtain reliable observation data for further regression analysis of the relationship between landscape metrics and RRES provision (Fig. 6). Therefore, the initial LULC map was reclassified into six LULC classes (agricultural land, residential area, green infrastructure, commercial/transportation/institutional (CTI), open space and water body) according to the ten administrative districts (Fig. 3). Then the plots were pre-stratified based on LULC classes and randomly distributed among the LULC classes and urban districts using Create Random Points tool in ArcMap 10.8.2 (Fig. 1).

Based on the field data, the i-Tree Eco model estimated the structural characteristics of the urban tree population. Using the structural traits of trees (including tree species, total tree height, tree height to crown base, crown width, and missing and total tree cover) along with location information and precipitation data, the RRES was calculated using the *Hydrology Effects of Trees module* in i-Tree Eco for the entire city and each LULC class and district. This module estimates the various components of RRES, including rainfall interception, storage, transpiration, and evaporation, contributing to runoff reduction (Wang et al. 2008; Hirabayashi 2013; Nowak 2021). The modified Rutter methodology was utilized to simulate the process of interception (Nowak 2021). Moreover, evaporation was simulated according to the research of Deardorff (1978) and Noilhan and Planton (1989). These estimates are process-based, meaning each process is simulated separately before being linked to other processes (Hirabayashi 2013; Nowak 2021). To assess the impact of urban trees on runoff, the module assumes two scenarios: the actual (current tree conditions) and hypothetical (without trees in the same area) scenarios. For both scenarios, hourly precipitation, interception, evaporation, transpiration, and potential evapotranspiration processes are simulated first, followed by the volume of annual surface runoff. The difference in generated surface runoff between the scenarios determines the annual net RRES. Due to the effects of trees by intercepting, storing, and evaporating rainwater, the actual scenario generates less runoff than the hypothetical one. The net avoided runoff is further summarized for each tree, species, and stratum. The methods and equations are detailed in Hirabayashi (2013) and Hirabayashi and Endreny (2016).

#### Analyzing the Urban Landscape Structural Pattern

To analyze the urban landscape structural pattern, the metrics related to the landscape structure of LULC classes were calculated using FRAGSTATS 4.0. The equations, ranges, and a short description of each landscape metric are summarized in Table 1.

**Table 1:** The detailed explanation of landscape structure-related metrics (Mcgarigal and Marks 1994; Rutledge 2003)

Landscape metrics	Symbol	Equation		Range	Description
Perimeter-Area Ratio	*PARA*	$PARA = \frac{{p_{ij}}}{{a_{ij}}}$	(1)	$PARA > 0$	Measures the extent of shape complexity without standardization to the simple Euclidean shape.
Shape Index	*SHP*	$SHP = \frac{{0.25\,p_{ij}}}{{\sqrt {a_{ij}} }}$	(2)	$SHP \ge 1$	Compares the complexity of the patch shape to the standard shape (square) of the same size.
Fractal Dimension Index	*FRAC*	$FRAC = \frac{{2\,\ln \left( {0.25\,p_{ij}} \right)}}{{\ln a_{ij}}}$	(3)	$1 \le FRAC \le 2$	Reflects the degree of patch shape complexity
Related Circumscribing Circle	*RCC*	$RCC = 1 - \left[ {\frac{{a_{ij}}}{{a_{ij}^s}}} \right]$	(4)	$0 \le RCC \le 1$	Measures the overall patch elongation
Contiguity Index	*CI*	$CI = \frac{{\left[ {\frac{{\mathop {\sum}\nolimits_{r = 1}^z {c_{ijr}} }}{{a_{ij}^ \ast }}} \right] - 1}}{{v - 1}}$	(5)	$0 \le CI \le 1$	Measures the spatial contiguity of cells in the grid cell patches (patch boundary configuration and patch shape)

${{{\boldsymbol{p}}}}_{{{{\boldsymbol{ij}}}}}$ : perimeter (*m*) of patch ij, ${{{\boldsymbol{a}}}}_{{{{\boldsymbol{ij}}}}}$: area (*m*^2^) of patch ij: area (*m*^2^) of the smallest circumscribing circle around patch ij, ${{{\boldsymbol{c}}}}_{{{{\boldsymbol{ijr}}}}}$: contiguity value for pixel r in patch ij, ${{{\boldsymbol{a}}}}_{{{{\boldsymbol{ij}}}}}^ \ast$: area of patch ij in terms of the number of cells, ${{{\boldsymbol{v}}}}$: sum of the values in a 3-by-3 cell template (13 in this case).

#### Analyzing the Relationships Between the Landscape Structural Pattern and the Provision of RRES

To model the relationship between landscape structure-related metrics and the provision of RRES, stepwise regression analysis was conducted using IBM SPSS 19 software. Stepwise regression analysis is the automated computational process using forward and backward selection techniques to obtain the optimal regression (Thatcher 2021). The model omits irrelevant variables and secures that independent variables are not correlated (Johnsson 1992; Thatcher 2021). Consequently, the landscape metrics were entered into the model as independent variables, while RRES was a dependent variable. P ≤ 0.05 and P ≥ 0.100 were applied to the entry and exclusion criteria. The model outlines which landscape metrics would better explain the RRES provision. This brought about the equation to estimate the RRES:

1 $$y_i = \beta _0 + \beta _1x_1 + \beta _2x_2 + \cdots + \beta _{n - 1}x_{n - 1} + \varepsilon _i$$

where *y*_*i*_ is the total annual RRES ($m^3/yr$) in the study area, *x*_1_…*x*_*n*-1_ are the landscape structure-related metrics (PARA, SHP, FRAC. RCC and CI), *β*_1_…*β*_*n*-1_ are the coefficients of city landscape metrics retained with P ≤ 0.05, $\beta _0$ is a constant of the model with P ≤ 0.05 and $\varepsilon _i$ is the error for the annual RRES.

The variation inflation factor (VIF) was also applied to assess the intervariable collinearity of the models obtained, where VIF < 10 states a lack of collinearity (Chatterjee and Hadi 2013). Scatter plots of observed versus predicted values of the total annual RRES were used to evaluate the goodness of fit for each model.

## Results

### Urban Tree Structure and the Corresponding RRES

**Fig. 4:**
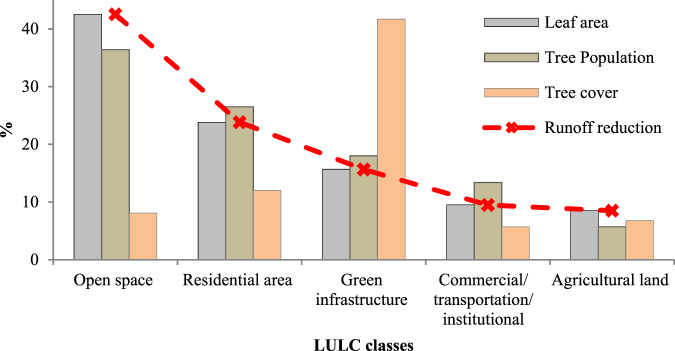
Comparison of urban trees’ structural traits and the runoff reduction among the LULC classes

The results showed that there were 1,927,566 trees (with a standard error of 12.3%), with a tree cover of 9.4% in the study area. Accordingly, they provided 8,373.04 km^2^ of leaf area (LA). Total LA was greatest for open spaces, followed by residential areas and GI. However, the GI, residential area and open space classes had the highest tree density, respectively (Fig. 4).

Among the administrative districts, the highest number of trees was observed in D6 (District 6), followed by D5 and D3. The total tree density was 79.33 trees ha^−1^, with the highest value in D10 (105 trees ha^−1^) (Table 2).

**Table 2:** Tree population summary by urban districts

Urban districts	Number of Trees	Percentage of Population	Tree Density (number ha^−1^)	Leaf Area (km^2^)	Leaf Area per hectare (km^2^ ha^−1^)
D1	141,550.00	7.34	91.77	601.49	0.3899
D2	205,958.00	10.68	99.33	859.67	0.4146
D3	232,665.00	12.07	83.62	961.09	0.3454
D4	196,119.00	10.17	79.77	848.67	0.3452
D5	238,648.00	12.38	75.59	1,098.14	0.3478
D6	490,798.00	25.46	68.56	2,189.08	0.3058
D7	229,349.00	11.90	79.44	991.74	0.3435
D8	32,822.00	1.70	85.75	131.69	0.3441
D9	49,333.00	2.56	61.32	237.00	0.2946
D10	110,324.00	5.72	104.94	454.46	0.4323
Total	1,927,566.00	100.00	79.33	8,373.04	0.3446

**Fig. 5:**
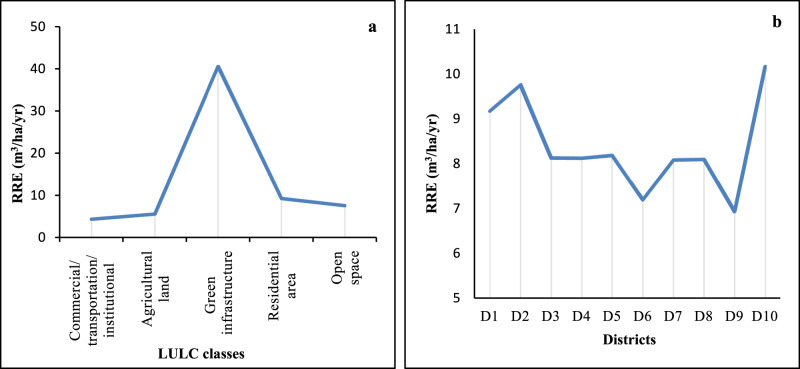
Runoff reduction efficiency (RRE) between the LULC classes (a) and districts (b). D1-D10 are urban districts

The results indicated that the trees reduced 196,854.15 m^3^ of runoff annually. Open spaces and agricultural land had the highest and lowest contribution to RRES, respectively. The majority of runoff (82%) was reduced by open spaces, residential areas, and GI at a total of 16,1425 m^3^ per year. This pattern is likely due to the different structural characteristics of urban trees in each LULC class (Fig. 4). GI class had the highest runoff reduction efficiency (40.51 m^3^ha^−1^yr^−1^), followed by residential areas, open spaces, agricultural land, and CTI (Fig. 5a).

The capacities of the different districts for runoff reduction indicated that D6 obtained the highest runoff reduction ratio (average of 28%). Districts 6, 5 and 7 were responsible for approximately half (51.1%) of total runoff reduction in the study area (Table 3). The results showed that districts’ runoff reduction efficiency (RRE) varies: D10 and D9 had the highest RRE with 10.16 and 6.92 (m^3^ha^−1^yr^−1^), respectively. The potential reason is that D10 and D9 have the greatest and lowest leaf area and tree number per hectare, respectively (Table 2).

**Table 3:** RRES by urban trees and its hydrological components within the urban districts

Urban Districts	Potential Evapotranspiration (m^3^yr^−1^)	Evaporation (m^3^yr^−1^)	Transpiration (m^3^yr^−1^)	Water Intercepted (m^3^yr^−1^)	Runoff Reduction (m^3^yr^−1^)
D1	950,925.46	77,479.02	395,132.67	77,796.61	14,141.27
D2	1,359,103.38	110,736.34	564,740.53	111,190.24	20,211.31
D3	1,519,439.43	123,800.12	631,364.07	124,307.57	22,595.68
D4	1,341,711.12	109,319.26	557,513.63	109,767.35	19,952.67
D5	1,736,116.24	141,454.40	721,398.56	142,034.22	25,817.89
D6	3,460,841.69	281,980.71	1,438,063.97	283,136.53	51,466.38
D7	1,567,898.50	127,748.44	651,499.99	128,272.08	23,316.31
D8	208,196.68	16,963.34	86,510.79	17,032.88	3,096.11
D9	374,684.20	30,528.33	155,690.40	30,653.46	5,571.95
D10	718,481.64	58,540.08	298,546.61	58,780.04	10,684.58
Total	13,237,398.33	1,078,550.05	5,500,461.22	1,082,970.97	196,854.15

### Landscape Structural Pattern of LULC Classes

Descriptive statistics, including mean, maximum, minimum, standard division, and variance, were calculated for all patches (LULC classes) within districts (Table 4 and Appendix 1).

**Table 4:** Descriptive statistics of landscape structure-related metrics

Landscape Metrics	Descriptive Statistics	LULC Classes				
		CTI	Agricultural land	Green infrastructure	Residential area	Open space
*RCC*	Mean	0.48	0.53	0.60	0.56	0.44
	Max.	0.53	0.62	0.74	0.59	0.52
	Min.	0.41	0.43	0.43	0.53	0.37
	Std.	0.04	0.06	0.10	0.02	0.06
	Var.	0.00	0.00	0.01	0.00	0.00
*CI*	Mean	0.45	0.84	0.48	0.73	0.16
	Max.	0.64	0.94	0.77	0.80	0.18
	Min.	0.27	0.75	0.31	0.66	0.12
	Std.	0.13	0.06	0.15	0.04	0.02
	Var.	0.02	0.00	0.02	0.00	0.00
*FRAC*	Mean	1.08	1.09	1.15	1.13	1.11
	Max.	1.10	1.11	1.20	1.14	1.15
	Min.	1.07	1.05	1.12	1.11	1.08
	Std.	0.01	0.02	0.03	0.01	0.02
	Var.	0.00	0.00	0.00	0.00	0.00
*PARA*	Mean	6,750.39	1,903.17	6,432.22	3,258.64	10,738.80
	Max.	9,118.51	3,133.51	8,802.72	4,119.78	11,383.50
	Min.	4,342.77	735.05	2,717.19	2,325.06	10,310.91
	Std.	1,704.02	782.88	2,155.84	566.97	359.81
	Var.	2,903,692.45	612,894.67	4,647,648.30	321,457.01	129,460.41
*SHP*	Mean	1.21	1.52	1.63	1.69	1.33
	Max.	1.37	1.74	1.92	1.91	1.51
	Min.	1.12	1.12	1.48	1.46	1.22
	Std.	0.08	0.20	0.14	0.15	0.10
	Var.	0.01	0.04	0.02	0.02	0.01

*Max.* Maximum, *Min.* Minimum, *Std.* Standard division, *Var.* Variance.

The results indicated that the LULC classes have different values of landscape structure-related metrics. The landscape metrics showed different maximum and minimum values, suggesting they all have unique insights to provide. The mean of *SHP* and *FRAC* for all patches was greater than 1, which means relative irregular, complex and convoluted patch shapes of LULC classes. All LULC patches had complex shapes because the relevant *PARA* values were high, indicating a deviation from the isodiametric shapes (larger edge for a given area). The *CI* results indicated that agricultural land had the highest patch connectedness (CI = 0.84), while the open space had the lowest patch contiguity (CI = 0.16). In total, the landscape metrics indicate that the LULC patches of the study area tended to be almost complex shapes.

### The Linkage Between Landscape Structural Pattern and RRES

**Fig. 6:**
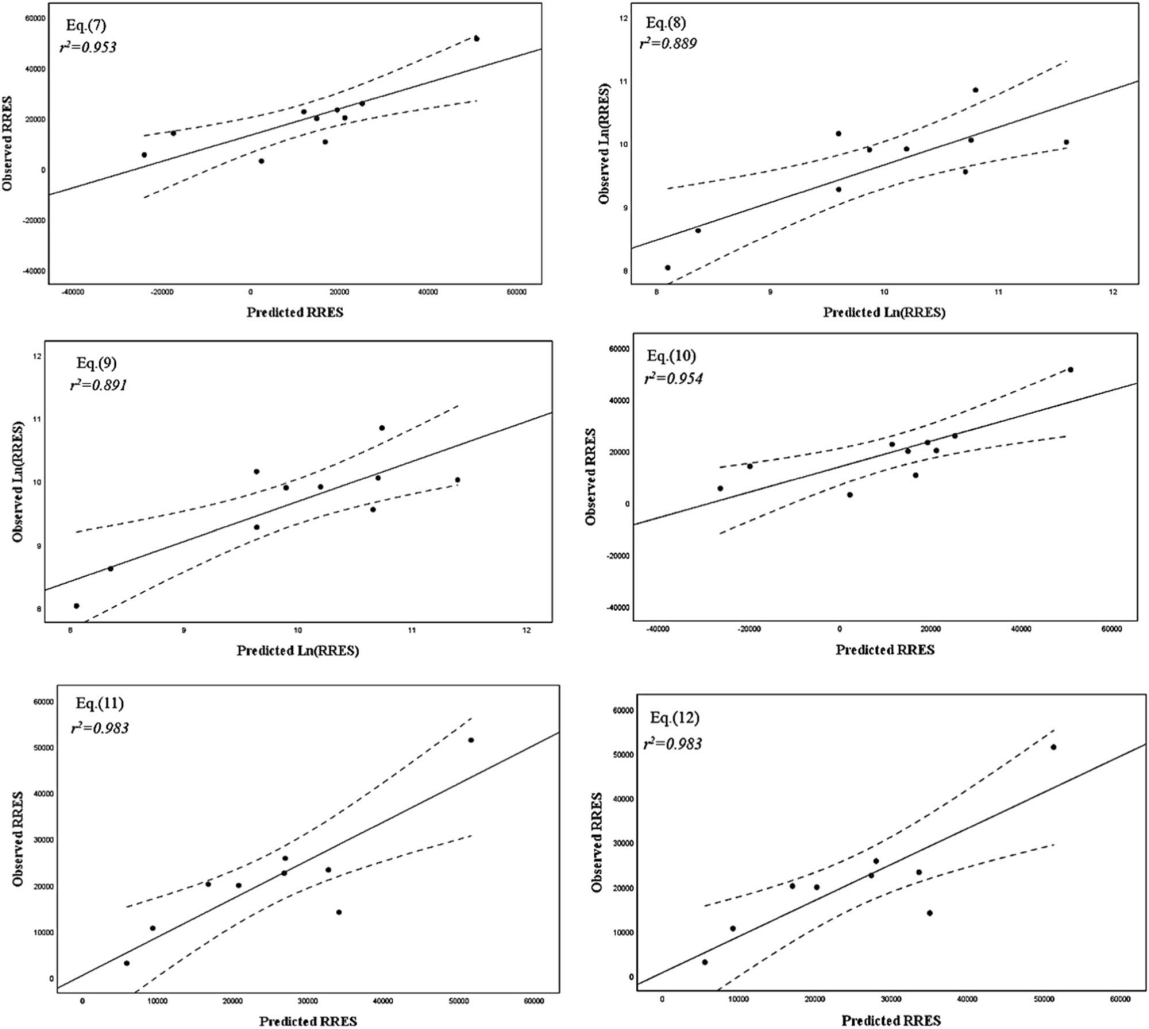
Predicted vs. observed values (m^3^yr^−1^) for RRES models using different landscape structure-related metrics

Multiple linear regression models were developed, explaining the RRES through landscape structure-related metrics measurements (Eqs. (2 to 7)). Other statistics for these models can be found in Table 5. The one-by-one relationships between observed and predicted RRES using landscape metrics are shown in Fig. 6.

2 $$RRES = - 601952.094 + 1224265.115\,R_{RCC} - 147837.089\,CTI_{RCC}$$

3 $$Ln\,RRES = - 0.048 + 18.791\,A_{RCC}$$

4 $$Ln\,RRES = 15.879 + 9.361\,Ln\left( {A_{RCC}} \right)$$

5 $$RRES = 372044.529 + 711165.569\,Ln\left( {R_{RCC}} \right) - 71587.563\,Ln\left( {CTI_{RCC}} \right)$$

6 $$RRES = - 135890.437 + 71403.049\,GI_{SHP} + 29249.765\,A_{SHP}$$

7 $$RRES = - 51322.510 + 123069.289\,Ln\left( {GI_{SHP}} \right) + 40982.882\,Ln\left( {A_{SHP}} \right)$$

where *RRES* is the annual runoff reduction provided by urban trees, *RCC* represents mean related circumscribing circle index for a given class of the LULC, *SHP* is the mean shape index for a given LULC type, *R* is the residential class, *A* is the agriculture class, *CTI* is the commercial/transportation/institutional class, and *Ln* represents natural logarithm.

**Table 5:** Statistics of regression models for the RRES using different landscape metrics

Model		Coefficients			*r*^*2*^	*t*	*p* value	Collinearity Statistics	
Number	Variable	*B*	Standard Error	Beta				Tolerance	VIF
Eq. (2)	Constant	−601,952.09	81,629.93		0.953	−7.374	0.005		
	*R*_*RCC*_	1,224,265.12	157,767.55	1.102		7.760	0.004	0.782	1.279
	*CTI*_*RCC*_	−147,837.09	46,013.46	−0.456		−3.213	0.049	0.782	1.279
Eq. (3)	Constant	−0.05	1.73		0.889	−0.028	0.979		
	*A*_*RCC*_	18.79	3.32	0.943		5.666	0.005	1.000	1.000
Eq. (4)	Constant	15.88	1.09		0.891	14.532	0.000		
	*Ln*(*A*_*RCC*_)	9.36	1.64	0.944		5.714	0.005	1.000	1.000
Eq. (5)	Constant	372,044.53	45,599.48		0.954	8.159	0.004		
	*Ln*(*R*_*RCC*_)	711,165.57	90,408.06	1.118		7.866	0.004	0.758	1.319
	*Ln*(*CTI*_*RCC*_)	−71,587.56	21,505.89	−0.473		−3.329	0.045	0.758	1.319
Eq. (6)	Constant	−135,890.44	12,629.30		0.983	−10.760	0.002		
	*GI*_*SHP*_	71,403.05	8,972.72	0.698		7.958	0.004	0.750	1.333
	*A*_*SHP*_	29,249.77	5,881.56	0.436		4.973	0.016	0.750	1.333
Eq. (7)	Constant	−51,322.51	6,197.89		0.983	−8.281	0.004		
	*Ln*(*GI*_*SHP*_)	123,069.29	14,622.22	0.710		8.417	0.004	0.782	1.279
	*Ln*(*A*_*SHP*_)	40,982.88	7,945.54	0.435		5.158	0.014	0.782	1.279

The stepwise regression modeling results indicated the structural pattern’s effects on the annual RRES. The relationships between the RRES and landscape metrics were highly significant (0.889 ≤ *r*^2^ ≤ 0.983) (Table 5). The results suggested that the total RRES could be predicted using the means of the two landscape metrics: the related circumscribing circle (0.889 ≤ *r*^2 ^ ≤ 0.954), and the shape index (*r*^2^ = 0.983) (Table 5), indicating these indexes explain 88.9 to 98.3% of the variation of RRES across the study area. Stepwise regression modeling determined the relevance of only four LULC classes, including residential areas, CTI, agricultural land, and GI (Eqs. 2 to 7). Furthermore, *PARA*, *FRA*, and *CI* indexes were not observed in the developed models.

Table 5 shows that the mean *RCC* indices of the residential and CTI patches (Eq. 2) statistically explain 95.3 % of the overall variations in the measures of RRES. The total RRES had a negative relationship with the associated related circumscribing circle index of CTI patches (*CTI*_*RCC*_) (Eq. 2), showing that the lower the CTI_RCC_ (i.e., the less narrow elongated the CTI patches are), the higher the RRES. It signifies that convoluting the shape of the CTI patches due to an increase in the RCC index would contribute to providing RRES in the city rather than elongation. Furthermore, the mean *RCC* for the residential area was positively correlated with the RRES. According to the *RCC* definition, the more narrow the elongated residential patches are, the greater the RRES in the city. This suggests that relatively narrow and elongated residential patches would play an important role in the RRES compared to relatively convoluted patches.

About 89% of the total variations in the RRES (Eqs. 3 and 4) were explained by the value of the *RCC* index of agricultural patches in the absence of any other metrics of LULC patches. Therefore, if the *A*_*RCC*_ Increases in the city, the RRES will increase significantly. This indicates that the RRES is influenced by agricultural area when the patches are more narrow and elongated in the city.

About 98% of the total variations in RRES (Eqs. 6 and 7) were significantly explained by a combination of the *GI*_*SHP*_ and *A*_*SHP*_ Hence, the shape index of GI and agricultural patches substantially affects RRES. According to the level of the model, the overall complexity of GI and agriculture patches may significantly explain the variations in RRES. Therefore, an increase in the shape index of the GI and agricultural patches (*GI*_*SHP*_ and *A*_*SHP*_) in the city could increase the RRES due to the higher shape irregularity of the GI and agricultural patches. Accordingly, only the modification of GI and agricultural land into square or nearly square (i.e., regularly shaped) patches would likely decrease the RRES throughout the city. The function can be deduced from the shape index of green infrastructure (*GI*_*SHP*_) and agriculture (*A*_*SHP*_) patches in the city’s landscape (Eqs. 6 and 7).

Moreover, using VIF, the intervariable collinearity of the models was assessed (Table 5). All models had VIFs smaller than 1.4, indicating a lack of collinearity.

## Discussion

Urban trees are recommended as an effective and complementary measure to alleviate the problem of urban stormwater runoff, improving urban sustainability (Mullaney et al. 2015; US EPA 2017; Lu and Wang 2021). To properly understand and utilize the capacity of urban trees for runoff mitigation, it is vital to obtain precise and reliable estimates of RRES. This work attempted to quantify the contributions of urban trees to runoff mitigation at the urban scale in Tabriz, Iran. The results indicated that urban trees are effective in mitigating runoff. They can reduce 196.85 × 10^3^ m^3^ of stormwater runoff annually. Different runoff reduction capacities have been observed due to the various urban LULC classes. The open spaces had shown the highest runoff reduction (Fig. 4). A potential reason is that open spaces tend to have the highest share of area (45.4%) in general and more leaf area and tree number in particular.

On the other hand, as regards runoff reduction efficiency (Fig. 5, a), GI, which covers the lowest area in the city (3.1%), has the highest efficiency due to the greatest leaf area per hectare and tree density (456 tree ha^−1^). This is also true for urban districts; the more tree density the district has, the more RRES was observed. This conclusion is reinforced by the fact that leaf area is one of the most important factors in runoff reduction process by urban trees (Nowak 2021). So, runoff reduction efficiency provides a better understanding of the potential of each LULC type and district in runoff reduction. Knowledge of the runoff reduction capacity of urban trees within LULC classes in different urban districts can contribute to proper management as local municipalities manage each district independently.

The effect of the GI and agricultural land in this study agrees with previous studies (Pace and Sales 2012; Mikulanis 2014; Nowak et al. 2017), identifying green spaces as the main source of runoff reduction. The comparison of urban tree traits and RRES across the cities (Table 6) indicates that Tabriz has a somewhat near-the-average tree number; however, the tree cover ranks among the lowest, exceeding only Phoenix, implying its trees are quite small and young. Estimated annual runoff reduction efficiency has ranged from 8.04 to 71.52 m^3^ per tree, within which Tabriz has the lowest value. Although tree characteristics may be the primary contributor to this low efficiency, as 78 % of the existing trees are not large enough to produce significant runoff reduction, the effects of rainfall (amount, duration, and pattern) could not be ignored on runoff reduction (Nytch et al. 2019). Despite the modest RRE in the study area, such a reduction in surface runoff can have considerable environmental benefits in addition to the significant reduction in stormwater management costs.

**Table 6:** Annual runoff reduction and trees for different cities

City	Tree			Runoff reduction			References
	Number (million)	Cover (%)	Number per hectare	(m^3^ yr^−1^)	(m^3^ tree^−1^ yr^−1^)	Efficiency (m^3^ ha^−1^ yr^−1^)	
Mesquite, U.S	2.09	24.4	174.7	855,858.00	0.409	71.52	(Pace and Sales 2012)
Houston, U.S	33.27	18.4	205.09	4,898,814.00	0.147	31.5	(Nowak et al. 2017)
Phoenix, Arizona, US	3.17	9	31.8	2,596,655.00	0.82	26.07	(Mikulanis 2014)
Plano, U.S	1.69	16.4	90.93	189,401.00	0.112	10.16	(PARD 2014)
London, UK	8.42	14	53	3,413,471.00	0.405	21.40	(Rogers et al. 2015)
Newport City, UK	0.26	12	54	87,900.00	0.338	18.10	(Buckland et al. 2020)
Oldham, UK	0.47	12	33	202,680.00	0.434	14.47	(Watson et al. 2017)
Tabriz, Iran	1.93	9.4	79.3	196,854.00	0.10	8.04	(this study)

The usefulness of the ES concept for landscape and ecosystem management depends on our knowledge of links between landscape structure and ES provision (Mitchell et al. 2013). Since HES provision can be either directly or indirectly affected by landscape structure (Chen et al. 2021; Yohannes et al. 2021), improving our knowledge of the interactions between landscape structure and RRES provision by integrating the concepts of landscape ecology and ES into urban hydrology helps effectively manage urban landscapes and resiliently maintain and enhance the sustainability of HES supply (Mitchell et al. 2013; Francis et al. 2022; Tran et al. 2022). However, the empirical understanding of how landscape structure impacts RRES provision remains limited. This gap limits our ability to manage urban landscape effectively for RRES. To bridge this gap, this study assessed the impacts of landscape structural patterns, particularly the shape of LULC patches, on RRES provision. The findings provided direct evidence that the shape of urban LULC patches significantly influences RRES capacity. This is consistent with previous studies demonstrating the importance of landscape structure in providing HES (Zhang et al. 2015; Li et al. 2020).

In doing so, we emphasize how LULC patches’ shape can mediate the RRES supply. To sum up the findings, it is noteworthy that only two of the five studied landscape structure-related metrics (shape and related circumscription circle metrics) have resulted in reliable models for predicting the provision of RRES. The results indicate that *SHP* and *RCC* metrics are the influential determinants of RRES and could be applied in RRES assessment. This is consistent with those of the previous study, which analyzed the links between flooding phenomena with landscape metrics on a larger scale using the same landscape metrics (Amiri et al. 2018).

The finding showed that the *RCC* metric for agricultural patches could be applied to develop the RRES prediction model. However, applying the RRES prediction models, which are based on the *RCC* metric for residential areas and CTI, could provide more reliable estimations to their users. Moreover, it is noteworthy that the more elongated the shape of the residential and agricultural patches, the greater the supply of RRES. Therefore, expanding agricultural and residential patches may only improve the capacity of trees to mitigate runoff if they have more elongated and narrower shapes, but an extended CTI with a more convoluted shape would be advantageous. However, regularity or irregularity in the shape of the GI and agricultural patches, specifically the degree by which their patches deviate from an iso-diametric shape as reflected by differences in shape index, was observed to be significantly related to the extent of RRES. The results showed that increasing the degree of shape irregularity in the GI and agricultural patches improves their contribution to runoff mitigation.

Even though the previous works (e.g., Buckland et al. 2020; Rogers et al. 2015; Watson et al. 2017) have demonstrated that urban trees in green spaces have a considerable impact on stormwater runoff; the results of this study suggest that, in addition to current GI cover, the shape of the GI patches should also be considered.

Our approach can help to understand the RRES provisioning mechanism better and provides useful information for the urban decision support system to improve the sustainable functionality of the landscape. We have found a strong influence of the structural pattern on RRES. While some of the relationships between landscape structure and HES have been outlined in previous research (Dobbs et al. 2014; Grafius et al. 2018; Karimi et al. 2021), this work expands our understanding of the influence of landscape structural pattern on the RRES.

The results showed evidence of support for the role of landscape structure in maintaining the RRES in urbanized areas now and into the future. Understanding the impacts of the structural pattern on ES is a significant research goal that provides a foundation for alternative landscape management, planning, and restoration strategies (Turner et al. 2013). These findings can contribute to improving urban landscape planning and management with respect to sustainable urban runoff reduction. This helps to cover the necessity of carrying out ES assessment in parallel with and according to the urban landscape planning process (Grunewald and Bastian 2015).

Assessing the impacts of different urban landscape plans on multiple dimensions particularly ES, is crucial for establishing optimal landscape strategies (Termorshuizen and Opdam 2009; Francis et al. 2022). Through integrating the i-Tree Eco measurements with conventional landscape structure metrics analyses, this research provides an explicit landscape metrics-based tool to describe variations in the RRES capacity. This provides a potential approach to evaluate the response of RRES to changes in the urban landscape structural pattern. Urban decision-makers and planners can use it to establish optimal spatial policies and assess the impacts of their landscape strategies on the capacity of tree to provide RRES. In fact, once the urban land use strategies are defined, the obtained regression models could be an easy-to-use way to rapidly and iteratively assess whether the proposed strategies will result in positive or negative changes in RRES. This helps to identify how to change the landscape to improve the RRES provision and is in line with the critical elopement of landscape planning which aims to maintain the functions of the landscape and ecosystem (Grunewald and Bastian 2015).

This study helps to link ES assessment and urban landscape planning, which initially have different focuses (Grunewald and Bastian 2015). We try to bridge a gap in the field of integrating ES into landscape ecology and spatial planning, which can ease dialog with different practitioners and decision-makers. Despite the growing body of literature on ES, it has not been fully integrated into urban landscape planning and decision-making (Anna Hermann et al. 2011). Some of the main questions that need to be answered are 1) how can the relationships between ES and landscape characteristics be quantified and modeled? and 2) what is the effect of landscape features on ES? (de Groot et al. 2010). One approach to cover these challenging questions is better understanding the interrelations between LULC and ES (Verburg et al. 2009). This study tries to answer these questions and aims to integrate the ES concept into urban landscape management, planning and decision-making by analyzing the interactions between RRES and structural characteristics of LULC. Integrating landscape concepts into ES helps the ES framework to convey the complex relationships of socio-ecological systems and resolve its operational gaps (Angelstam et al. 2019).

This paper also helps to cover one of the main research directions of the "ES at the landscape scale" (Müller et al. 2010) by providing a suitable methodology to apply ES at the landscape scale and integrating ES in landscape analysis. This study contributes to the existing body of literature (Bastian 2001; Syrbe and Walz 2012; Babí Almenar et al. 2018), which advocate expanding the landscape ecology paradigm and highlighting the necessity for making an appropriate foundation for the resolution of urban planning subjects through analyzing the linkage between landscape structure and ES.

Although using the i-Tree Eco model to estimate RRES offers distinct benefits, including utilizing locally gathered field data, process-based hydrology estimations and modeling, and eco-hydrology of trees, it also has uncertainties and limitations. These drawbacks stem from simplifying (sub)surface hydrology to reflect the effects of urban trees, excluding of changing amounts of impervious cover, dismissing the impacts of the various spatial configuration of trees or other LULC types and applying default soil and hydrologic parameters (Hirabayashi 2013; Nowak 2021). Future research is required to help overcome these uncertainties and limitations.

Another limitation of this study is that it has focused on analyzing the effects of landscape structural patterns in an urban area with the varying terrain and topographic and hydrologic gradients, which might be considered to identify the impact of these variables.

To improve the knowledge of how landscape structure influences HES, future works are needed to consider additional biophysical, cultural, and social drivers at different spatial and temporal scales, as these factors determine ES distribution (Eigenbrod 2016). Further attempts are needed to study the impacts of landscape structure on multiple ES at once (ES bundles) and other dimensions of the ES delivery process. Comprehensive scenario analysis of future changes in rainfall, tree characteristics, landscape structure, LULC, and subsequently in RRES is required for long-term sustainable urban planning. Furthermore, analysis of the impacts of the other aspects of landscape, such as composition and connectivity on RRES using other landscape metrics can be considered in additional research.

## Conclusions

This paper provides the empirical basis to evaluate the hypothesis that urban landscape structural pattern impacts the REES provision. First, we provided the theoretical fundaments that suggest the landscape structure would affect the supply of HES and how common research concentrates on the links between landscape structure and HES. Second, by developing a new approach, we brought empirical evidence of how urban landscape structure affects RRES, which is required to manage and model RRES provision across urban landscapes accurately.

The idea for this work was due to the absence of empirical evidence on the relationship between landscape structure and RRES. This paper provided an explicit location-based estimation tool based on landscape metrics to describe variations in the RRES.

This study revealed the significant influence of the spatial shape of landscape on RRES and showed linear responses of the RRES to landscape metrics: shape and related circumscribing circle. Specifically, consistent with the shape-function relationship principle, we argue that the landscape structural pattern will significantly mediate the provision of RRES.

Our approach made it possible to predict the effects of changes in landscape structure on providing RRES. The findings have indicated that a change in the shape of the LULC due to the alteration of the structural attributes and landscape metrics of the LULC would cause a change in the runoff reduction capacity of trees as a process.

The findings would help urban environmental managers and policymakers better understand the importance of landscape structure when thinking about improving runoff mitigation capacity and, consequently, establishing proper LULC strategies through optimizing landscape metrics that result in positive changes in the supply of RRES. The landscape structure metrics could be served as capable and cost-effective indicators to assess RRES and monitor changes in RRES provision produced by several urban plans, such as a masterplan.

This work provides practical information for urban spatial planning by incorporating ES concept and landscape ecological perspective. The results could improve urban plans by considering landscape structure in the RRES supply.

This research helps to overcome the lack of a coherent and integrated approach to ES assessment at the level of methods. The findings contributed to an evolving body of knowledge on the relationship between landscape structure and ES provision and help to incorporate landscape structure into ES framework. The findings help to pave the way for expanding the urban landscape ecology paradigm and provide an appropriate foundation for the resolution of urban planning subjects through analyzing the linkage between landscape structure and RRES.

We suggest that this work may give a flexible approach with the potential to advance the application of the ES concept in practice for sustainable urban stormwater management and help to improve current tools and approaches. As the ES concept is increasingly integrated into urban decision-making and planning processes, this research contributes to a better understanding of the provision of ES on the landscape scale.

Expanding the approach to other cities and ES can illuminate and improve the capacity to identify ecological value in terms of ES provision and emphasize ES’s essential structural factors specific to each landscape.

## Appendix 1 The landscape structure-related metrics distribution statistics for LULC classes within each district

**Table:**

Urban districts	Landscape metrics																								
	*CRC*					*CI*					*FRAC*					*PARA*					*SHP*				
	CTI	Agricultural land	GI	Residential area	Open space	CTI	Agricultural land	GI	Residential area	Open space	CTI	Agricultural land	GI	Residential area	Open space	CTI	Agricultural land	GI	Residential area	Open space	CTI	Agricultural land	GI	Residential area	Open space
D1	0.47	0.57	0.64	0.53	0.38	0.35	0.94	0.31	0.73	0.12	1.08	1.10	1.19	1.12	1.10	8,033.30	735.05	8,746.02	3,298.51	11,383.50	1.16	1.61	1.72	1.68	1.26
D2	0.51	0.54	0.43	0.57	0.37	0.48	0.89	0.34	0.80	0.15	1.08	1.09	1.12	1.11	1.08	6,204.21	1,250.72	8,802.72	2,325.06	10,797.28	1.18	1.50	1.52	1.54	1.25
D3	0.47	0.62	0.62	0.56	0.40	0.38	0.90	0.60	0.71	0.15	1.07	1.11	1.14	1.13	1.09	7,578.55	1,274.98	4,608.95	3,422.23	10,634.94	1.14	1.63	1.61	1.75	1.22
D4	0.41	0.53	0.57	0.55	0.45	0.29	0.84	0.52	0.66	0.15	1.07	1.10	1.12	1.14	1.12	8,939.97	1,937.47	5,693.92	4,119.78	10,951.68	1.12	1.69	1.50	1.86	1.32
D5	0.52	0.51	0.58	0.57	0.46	0.59	0.81	0.43	0.76	0.18	1.09	1.08	1.17	1.12	1.13	5,008.45	2,359.80	7,489.43	2,863.24	10,619.74	1.27	1.54	1.65	1.53	1.48
D6	0.49	0.58	0.74	0.59	0.52	0.50	0.75	0.55	0.67	0.18	1.08	1.11	1.20	1.13	1.14	6,078.83	3,133.51	5,177.65	3,986.69	10,310.91	1.21	1.74	1.92	1.68	1.41
D7	0.49	0.57	0.65	0.57	0.47	0.40	0.90	0.77	0.70	0.17	1.09	1.10	1.13	1.13	1.12	7,318.64	1,158.45	2,717.19	3,677.71	10,462.75	1.19	1.61	1.70	1.66	1.35
D8	0.53	0.43	0.71	0.56	0.44	0.59	0.77	0.47	0.76	0.18	1.10	1.07	1.17	1.13	1.10	4,880.62	2,745.88	5,870.29	2,848.75	10,360.38	1.29	1.23	1.48	1.83	1.30
D9	0.51	0.45		0.53	0.51	0.64	0.80		0.77	0.17	1.09	1.09		1.11	1.15	4,342.77	2,487.10		2,787.89	10,619.09	1.37	1.51		1.46	1.51
D10	0.43	0.51	0.47	0.56	0.38	0.27	0.82	0.32	0.73	0.13	1.08	1.05	1.14	1.14	1.10	9,118.51	1,948.72	8,783.82	3,256.55	11,247.70	1.13	1.12	1.58	1.91	1.23

*CTI* Commercial/ Transportation /institutional, *GI* Green infrastructure.
